# Comparison of the clinical outcomes between proximal femoral nail anti-rotation with cement enhancement and hemiarthroplasty among elderly osteoporotic patients with intertrochanteric fracture

**DOI:** 10.1186/s12891-024-07414-0

**Published:** 2024-04-15

**Authors:** Xiang Zhou, Tao Chen, Yu-lin Jiang, De-bin Chen, Zhi-yong Tian

**Affiliations:** Department of Articular and Traumatic Orthopedic Surgery, the Fourth People’s Hospital of Guiyang, 91# Jiefang west Road, Guiyang, 550001 China

**Keywords:** Proximal femoral nail anti-rotation, Cement-enhanced internal fixation, Hemiarthroplasty, Intertrochanteric fracture, Osteoporotic

## Abstract

**Background:**

The proximal femoral nail anti-rotation (PFNA) with cement enhancement enhances the anchorage ability of internal fixation in elderly with osteoporotic intertrochanteric fracture. However, whether it is superior to hemiarthroplasty is still controversial. The present study aimed to determine which treatment has better clinical outcomes among older patients.

**Methods:**

We retrospectively analyzed 102 elderly patients with osteoporosis who developed intertrochanteric fractures and underwent PFNA combined with cement-enhanced internal fixation (*n* = 52, CE group), and hemiarthroplasty (*n* = 50, HA group) from September 2012 to October 2018. All the intertrochanteric fractures were classified according to the AO/OTA classification. Additionally, the operative time, intraoperative blood loss, intraoperative and postoperative blood transfusion rates, postoperative weight-bearing time, hospitalization time, Barthel Index of Activities Daily Living, Harris score of hip function, visual analog (VAS) pain score, and postoperative complications were compared between the two groups.

**Results:**

The CE group had significantly shorter operative time, lesser intraoperative blood loss, lower blood transfusion rate, and longer postoperative weight-bearing time than the HA group. The CE group had lower Barthel’s Index of Activities of Daily Living, lower Harris’ score, and higher VAS scores in the first and third months after surgery than the HA group, but no difference was observed between the two groups from 6 months to 12 months. There was no significant difference in the total post-operative complications between the two groups.

**Conclusion:**

The use of PFNA combined with a cement-enhanced internal fixation technique led to shorter operative time and lesser intraoperative blood loss and trauma in elderly patients as compared to HA.

## Introduction

Intertrochanteric fractures, which are more common in the elderly population, are mostly low-energy injuries that are usually accompanied by unstable comminuted fractures [1–3]. In the aging population, the incidence of intertrochanteric fractures increases each year, with a high complication, morbidity, and mortality rates due to advanced age, osteoporosis, and presence of combined underlying disease [4–7]. There is a consensus that early surgical treatment of intertrochanteric fractures reduces the incidence of fracture complications as well as morbidity and mortality [8]. Among the various surgical treatments, the proximal femoral nail anti-rotation (PFNA) intramedullary fixation system has become the primary option due to its low surgical trauma, minimal intraoperative blood loss, and excellent biomechanical advantages [9]. Although using PFNA internal fixation for elderly patients with osteoporosis has some benefits, cases of surgical failure still exist since helical blades may penetrate the head or neck, bend, or separate from the shaft [10].

To address this PFNA shortcoming, clinical studies have shown the advantage of using cement augmentation techniques to enhance the structural strength of the spiral blade–cement–bone interface complex to resist internal fixation displacement and prevent reoperation [11]. However, this technology is not yet widely used in clinical practice. Recently, hemiarthroplasty (HA) techniques have been successfully used to treat elderly patients with intertrochanteric fractures, and they have shown similar clinical outcomes as arthroplasty in patients with femoral neck fractures, especially in terms of early weight-bearing. Clinically, HA has been used as an alternative surgical option for elderly patients with osteoporosis [12]. HA offers advantages in early recovery of weight-bearing status and rapid restoration of limb function among elderly patients suffering from intertrochanteric fracture complicated with severe osteoporosis and having poor prognosis after internal fixation, short life expectancy, and poor stability of comminuted fractures [12]. However, the surgical risks are relatively higher because arthroplasty involves more significant surgical trauma and greater physiological disruption among elderly patients.

Therefore, the surgical options for elderly patients with osteoporotic intertrochanteric fractures are controversial [13, 14]. In the present study, we retrospectively compared the clinical data of PFNA combined with cement-enhanced internal fixation (CE group) and hemiarthroplasty (HA group) in the treatment of patients with osteoporotic intertrochanteric fractures in the advanced age and the complication rate of the related surgical treatment to provide a basis for finding the better option for clinical treatment.

## Methods

### Study design and patients

We retrospectively analyzed the medical records of 102 osteoporotic elderly patients with intertrochanteric fractures who had been treated at the Department of Trauma, Guiyang Fourth People’s Hospital, China, from September 2012 to October 2018. The Guiyang Fourth People’s Hospital’s clinical research ethics committee provided approval for conducting the present study. Both the guidelines stipulated in the Helsinki Declaration and Good Clinical Practice’s rules were followed in this investigation. In this study, 52 patients received PFNA combined with cement-enhanced internal fixation (CE group) and 50 patients underwent hemiarthroplasty (HA group).

**Fig. 1:**
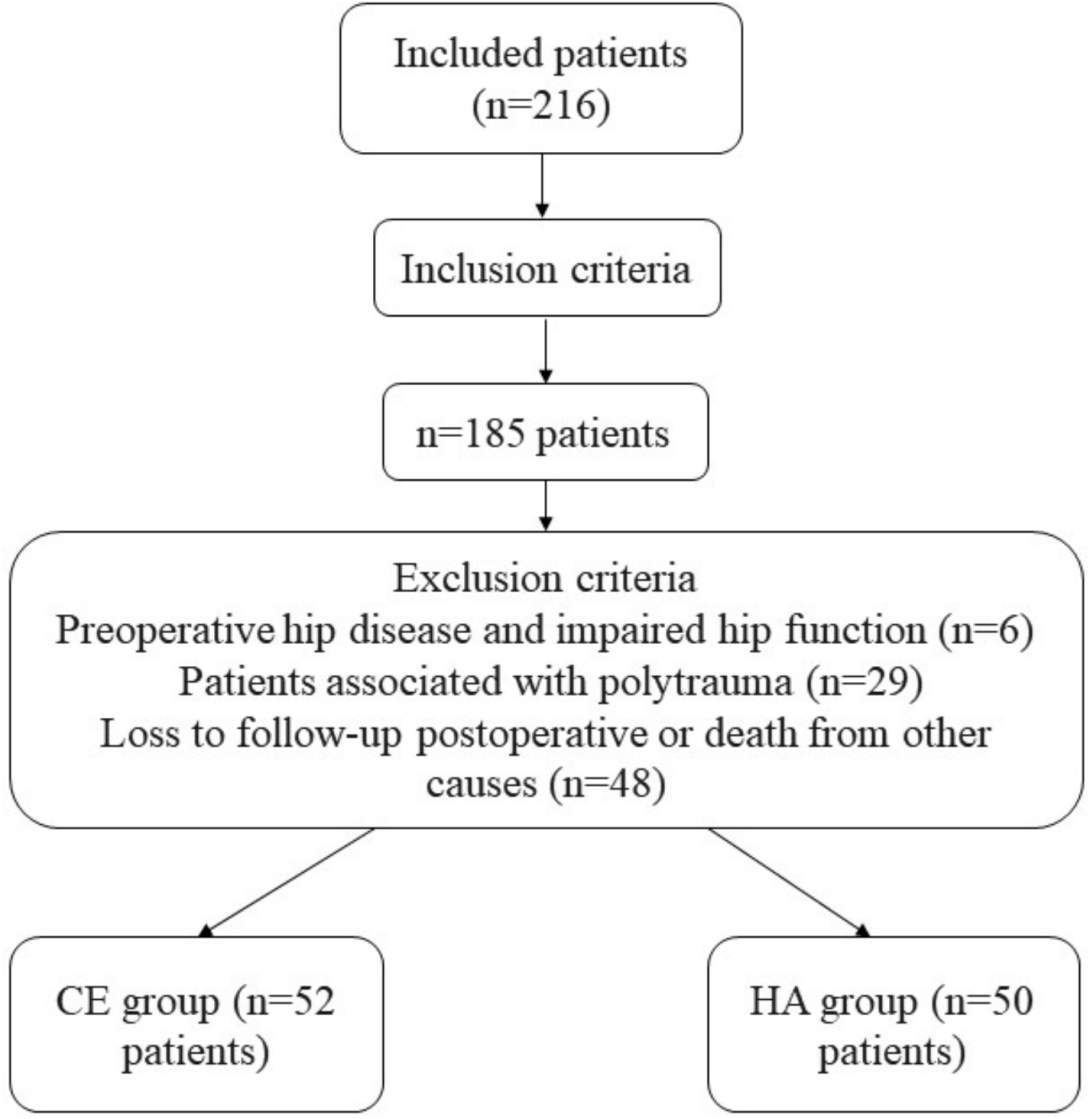
Flow chart of case screening

The inclusion criteria were as follows: (1) patients with intertrochanteric fractures; (2) patients aged < 65 years who had severe osteoporosis with a bone density T value of − 2.5 as determined by dual-energy X-ray absorptiometry (DXA) in the anteroposterior and lateral hips and L1–L4 vertebrae; (3) patients not considering the removal of the internal fixation; (4) patients who can tolerate and voluntarily undergo the operation; and (5) patients who can be followed up for 12 months after the operation. The exclusion criteria were (1) preoperative hip disease and impaired hip function; (2) pathological fractures; (3) with polytrauma; and (4) loss to follow-up during the postoperative period or death from other causes. The flow chart of case screening is shown in Fig. 1.

### Surgical procedures

The decision on the surgical strategy was reached through a comprehensive risk–benefit assessment, considering the preferences and wishes of patients and their family members. The operations in the HA and CE groups were performed by two experienced senior surgeons in the same team.

In the CE group, the patient is placed in a supine position and asked to lie down on the orthopedic traction bed after the combined spinal and epidural anesthesia has taken effect. The hip joint on the healthy side flexed, slightly abducted, and externally rotated while the surgical limb is straightened. Then, traction is applied with internal retraction and internal rotation of approximately 15° for surgical limb closed reduction. According to the Francisco intertrochanteric fracture reduction quality score [15], if the traditional reduction methods cannot provide a satisfactory result, a tiny incision is needed for the additional reduction procedures for some complex cases (AO/OTA 31-A2.3 or 31-A3) during the operation, such as clamping or levering. The surgeon sterilized the surgical area when the fracture was reduced as appropriated in the anteroposterior and lateral positions under fluoroscopic guidance. Then, an approximately 3 cm longitudinal incision is made from 1 cm above the greater trochanter’s apex. After palpating the top of the greater trochanter, an opening is made at the front and middle 1/3 between the apexes of the greater trochanter. Then, a PFNA nail with an appropriate size is inserted into the femoral marrow cavity. Under fluoroscopic guidance, we will drill along the femoral neck–head guide wire until 5–10 mm below the femoral head to avoid penetrating the cartilage into the femoral head to the hip joint and a spiral blade with a suitable length is prepared as backup after measuring the depth that the case need. Then, 5 mL of low-viscosity bone cement (Heraeus Low Viscosity 40 g/box) is injected into the femoral head with a syringe connected to two medical suction tips (China Henan Camel-man Best Medical Equipment Co., Ltd., the model I, with an external diameter of 6.0 mm, an internal diameter of 3.5 mm, and a suction head length of 19.8 mm). Then, a spiral blade with suitable length is placed into the appropriate position on the femoral head, and it is tightened and pressurized after the bone cement had solidified.

In the HA group, after the combined spinal and epidural anesthesia has taken effect, the patient is placed in the lateral position. Through the posterior–lateral approach, the posterior joint capsule is incised; then, the hip joint and intertrochanteric fragments were exposed. These fragments at the attachment point of the gluteus medius muscle of the greater trochanter should be preserved and fixed with wires or non-absorbable sutures after reduction. The femoral neck was osteotomized at approximately 1 to 1.5 cm above the lesser trochanter, and the femoral head was removed after measuring its size. To repair the femoral gluteus medius muscle attachment point, the hip is gently flexed, and the fracture is relocated in the trochanteric region and fixed firmly with a wire or non-absorbable sutures. An adapted type of biotype elongated femoral stem prosthesis and bipolar artificial femoral head was implanted after reducing the hip joint and testing the artificial hip joint stability by closing the incision.

### Peri-operative management

The skin of the operative area was cleaned. Prophylactic intravenous antibiotics were administered 30 min before the surgery. A subcutaneous injection of calcium natriuretic heparin was administered 8 h after surgery to prevent deep vein thrombosis. After the intervention of a staff from the rehabilitation department on the first postoperative day, the functional rehabilitation exercise of the affected limb was carried out according to the rehabilitation process of hip fracture.

### Postoperative follow-up method and observation index

The operative time, intraoperative blood loss, intraoperative and postoperative blood transfusion rates, postoperative weight-bearing time, average hospitalization days, Barthel Index of activities of Daily Living, Harris score of hip function, visual analog score (VAS) for pain, and postoperative complications at the 1st, 3rd, 6th and 12th months after the surgery were recorded in both groups.

### Statistical analysis

The statistical software used was SPSS 22.0, and the data of measurement information met normal distribution, expressed by mean ± standard deviation, and the independent sample t-test was used for comparison between groups. The data did not meet normal distribution, expressed by median (interquartile range) (M [P25% (P1), P75% (P3)]), and the Mann–Whitney U test was used for comparison between groups, and the Wilcoxon-signed rank test was used for comparison between groups. The count data were expressed by n (%), and the chi-square test was used for comparison between groups, and the significant difference was set as *p* < 0.05.

## Results

### Clinical characteristics of the study participants

**Fig. 2:**
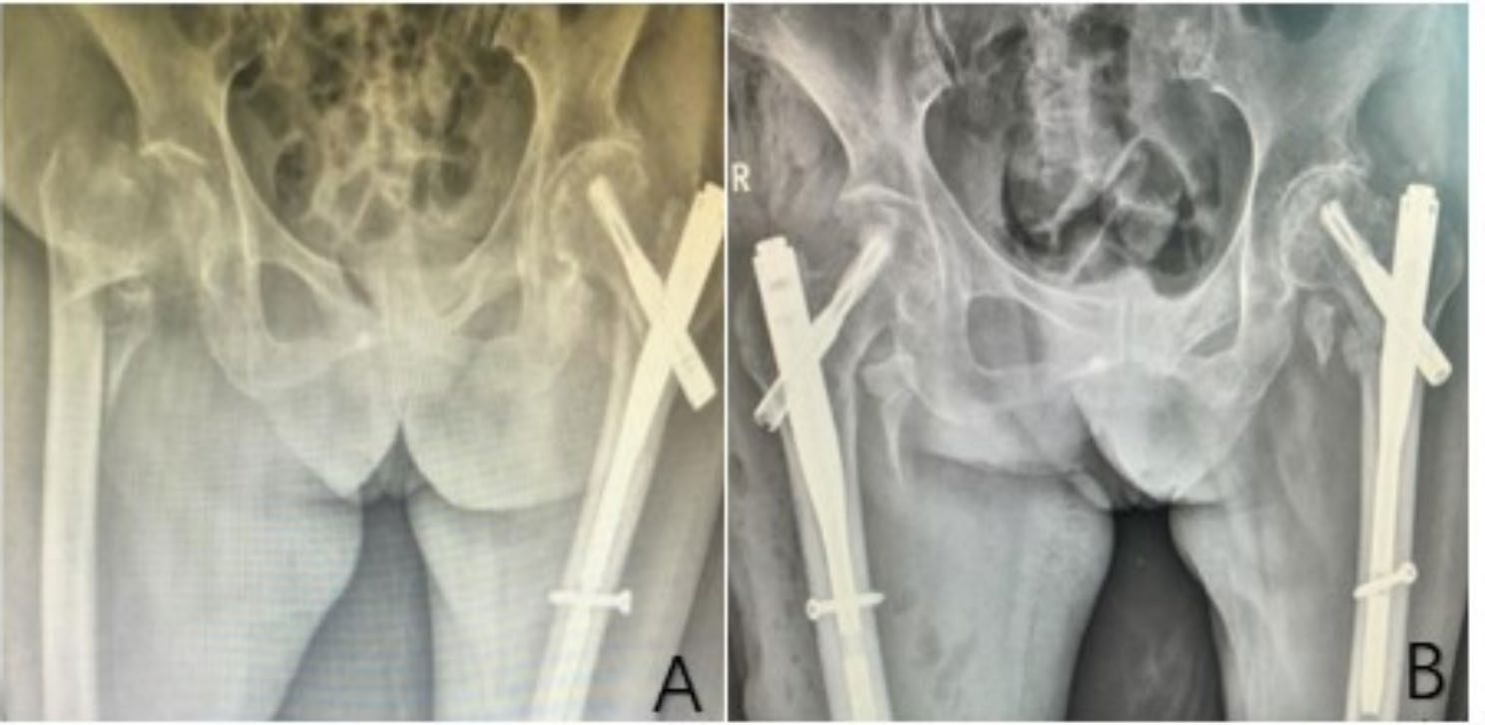
Preoperative and postoperative X-ray imaging of patient in the CE group. A-B: An 82-year-old female patient treated with PFNA cement-enhanced internal fixation technique for a right femoral intertrochanteric fracture (AO/OTA 31A2.3) on the preoperative image (**A**) and 6-month postoperative follow-up image (**B**) showed the right femoral intertrochanteric fracture varus reduction and union

**Fig. 3:**
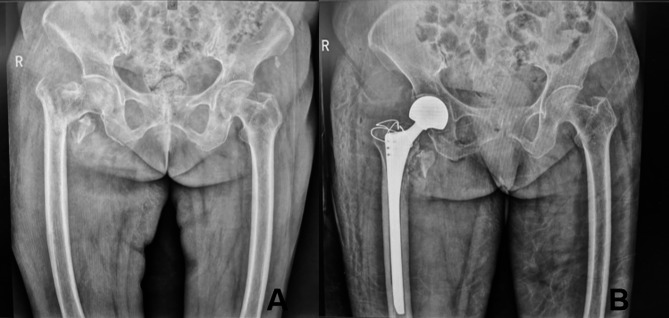
Preoperative and postoperative X-ray images of a patient in the HA group. **A**: The preoperative image (**A**) of an 86-year-old female patient showed a right femoral intertrochanteric fracture (AO/OTA 31A2.3), which was treated with artificial femoral head replacement. **B**: At 6 months postoperatively, the follow-up imaging results (**B**) showed a good position of the right artificial femoral head prosthesis and reliable fixation by wire binding of the greater trochanteric gluteus medius attachment point

Altogether, 102 patients were finally retrospectively analyzed in the study, and all patients had a mean follow-up duration of 18 months (12–24 months). There were no significant differences in age (78.00 ± 6.95 years vs. 80.04 ± 6.39 years), sex (male/female: 8/44 vs. 10/40), BMI (27.26 ± 2.47 kg/m^2^vs. 26.47 ± 2.35 kg/m^2^), bone density (− 3.1 [− 4.05, − 2.65] vs. −3.4 [− 3.8, − 3]), AO/OTA classification (31-A1/31-A2/31-A3: 1/44/7 vs. 1/43/6), age-adjusted CCI (4.115 ± 1.06 vs. 4.4 ± 0.83), ASA score (III/IV: 25/27 vs. 22/28) and comorbid diseases (cardiovascular/respiratory/neurologic disease/diabetes/urinary: 17/4/5/15/2 vs. 18/6/4/17/3) between the two groups (*p* > 0.05) (Table 1). According to the analysis of the follow-up imaging results, all the fractures were union in the CE group, and the cervical stem and anterior tilt angles were significantly corrected in both the CE and HA groups as compared to the preoperative period (Figs. 2 and 3).

**Table 1:** General comparison between the two groups

Group		CE	HA	χ^2^/t/z	P
Cases		52	50		
Age (years)		78.00 ± 6.95	80.04 ± 6.39	−1.542	0.126
Gender, n(%)	Male	8 (15.4)	10 (20.0)	0.374^a^	0.541
	Female	44 (84.6)	40 (80.0)		
BMI		27.26 ± 2.47	26.47 ± 2.35	1.641	0.104
Bone density, M(P1, P3)		−3.1 (− 4.05, − 2.65)	−3.4 (− 3.8, − 3)	−1.473	0.141
**AO/OTA classification, n(%)**					
	31-A1	1(1.9)	1(2.0)	0.049 ^a^	0.976
	31-A2	44(84.6)	43(86.0)		
	31-A3	7(13.5)	6(12.0)		
Age-adjusted CCI		4.115 ± 1.06	4.4 ± 0.83	1.504	0.136
ASA score, n(%)	III	25(48.1)	22(44.0)	0.171 ^a^	0.679
	IV	27(51.9)	28(56.0)		
**Comorbidities diseases, n(%)**					
	Cardiovascular	17(32.7)	18(36.0)	0.124 ^a^	0.725
	Respiratory	4((7.7)	6(12.0)	0.159^b^	0.690
	Neurologic disease	5(9.6)	4(8.0)	0.004 ^b^	0.951
	Diabetes	15(28.8)	17(34.0)	0.315 ^a^	0.575
	Urinary	2(3.8)	3(6.0)	0.002 ^b^	0.964

*Abbreviations* M (P1, P3): median (P25%, P75%), CE: cement-enhanced internal fixation, HA: hemiarthroplasty, BMI: body mass index, CCI: Charlson’s comorbidity Index, ASA: American Society of Anesthesiologists physical status classification system

^a^Chi-squared test, ^b^Using chi-squared test with Yates’ correction

### Comparison of the perioperative indicators between the two groups

The differences in operative time, intraoperative blood loss, intraoperative and postoperative blood transfusion rates, and postoperative weight-bearing time between the two groups were statistically significant (*p* < 0.05), with the data being better in the CE group than in the HA group. However, the remaining parameters between the two groups were not statistically significant (*p* > 0.05) (Table 2).

**Table 2:** Comparison of the relevant parameters during surgery between the two groups

Group	CE	HA	z	P
cases	52	50		
Operating time (min), M(P1, P3)	60 (45, 70)	85 (75, 90)	−6.813	< 0.001
Intra-operative BL (ml), M(P1, P3)	100 (100, 175)	200 (200, 300)	−6.566	< 0.001
Blood transfusion (ml), M(P1, P3)	0 (0, 0)	0 (0, 100)	−3.694	< 0.001
Inpatient days (d), M(P1, P3)	10 (8, 13.5)	10 (9, 13)	−0.609	0.542
Post-operative WBT (d), M(P1, P3)	6 (5, 9.5)	2 (2, 3)	−7.774	< 0.001

*Abbreviations* M (P1, P3): median (P25%, P75%), CE: cement-enhanced internal fixation; HA: hemiarthroplasty; BL: blood loss; WBT: weight bearing time

### Comparison of the changes in the Barthel Index of Activities of Daily Living ability between the two groups

After comparing the results between groups, the differences in the Barthel Index of Activities of Daily Living between the two groups at 1 and 3 months after surgery were statistically significant (*p* < 0.05), and both data were better in the HA group than in the CE group. There was no statistically significant difference in the data between the two groups at 6 and 12 months postoperatively (*p* > 0.05). The Barthel Index of Activities of Daily Living in both the CE and HA groups were significantly increased with increasing time from postoperative 1 month to 12 months (*p* < 0.05) (Table 3).

**Table 3:** Comparison of the changes in Barthel index of activities of daily living, Harris scores and VAS scores between the two groups

Group	Cases	Post-operative 1 month	Post-operative 3 months	Post-operative 6 months	Post-operative 12 months
**Barthel Index**					
CE, M(P1, P3)	52	35 (35, 35)	45 (40, 45)^a^	80 (75, 85)^ab^	85 (80, 85)^abc^
HA, M(P1, P3)	50	45 (40, 45)	70 (70, 75)^a^	80 (80, 85)^ab^	85 (80, 85)^abc^
z		−8.004	−8.804	−0.099	−1.092
*p*		< 0.001	< 0.001	0.921	0.275
**Harris scores**					
CE	52	45.69 ± 5.37	61.83 ± 5.90^a^	78.69 ± 4.98^ab^	84.31 ± 5.13^abc^
HA	50	61.92 ± 3.45	76.94 ± 4.26^a^	78.98 ± 3.91^ab^	84.00 ± 3.10^abc^
t		−18.066	−14.783	−0.323	0.365
*p*		< 0.001	< 0.001	0.747	0.716
**VAS scores**					
CE, M(P1, P3)	52	5 (4, 5)	2.5 (2, 3)^a^	2 (1, 2)^ab^	0 (0, 1)^abc^
HA, M(P1, P3)	50	3 (3, 3)	2 (2, 3)^a^	2 (1, 2)^ab^	1 (0, 1)^abc^
z		−6.598	−2.699	−0.04	−0.627
*p*		< 0.001	0.007	0.968	0.530

*Note* The data were expressed by M (P1, P3) or mean ± standard deviation. M (P1, P3): median (P25%, P75%), CE: cement-enhanced internal fixation, HA: hemiarthroplasty

^a^*p* < 0.05 vs. Post-operative 1 month; ^b^*p* < 0.05 vs. Post-operative 3 months; ^c^*p* < 0.05 vs. Post-operative 6 months

### The changes in Harris scores between the two groups

The differences in Harris scores between the two groups at 1 and 3 months postoperatively were statistically significant (*p* < 0.05), and both values were better in the HA group than in the CE group. There was no statistically significant difference in the data between the two groups at 6 and 12 months postoperatively (*p* > 0.05). The Harris scores of both the CE and HA groups were significantly increased with increasing time from post-operative 1 month to 12 months (*p* < 0.05) (Table 3).

### The changes in VAS scores between the two groups

The differences in VAS scores between the two groups at 1 and 3 months postoperatively were statistically significant (*p* < 0.05), and both scores were lower in the HA group than in the CE group. There was no statistically significant difference in the data between groups at 6 and 12 months postoperatively (*p* > 0.05). The VAS scores of both the CE and HA groups were significantly reduced with increasing time from post-operative 1 month to 12 months (*p* < 0.05) (Table 3).

### The post-operative complications between the two groups

There was no significant difference in the total post-operative complications between the two groups (*p* > 0.05). Among the CE group, one patient had a wound infection, 2 patients had deep vein thrombosis (DVT), 4 patients had cardiovascular complications, 2 patients had respiratory complications, and 2 patients had urinary complications, but no one had cut-out failure or fracture collapse. Among the HA group, 6 patients had postoperative aseptic loosening, 1 patient had a wound infection, 3 patients had DVT, 2 patients had cardiovascular complications, 3 patients had respiratory complications, and 1 patient had urinary complications. There was no significant difference between the two groups in terms of complications such as wound infection, lower limb DVT, and postoperative cardiovascular, respiratory and urinary complications (*p* > 0.05) (Table 4).

**Table 4:** Comparison of the post-operative complications between the two groups

Group	CE	HA	χ^2^	P
Cases	52	50		
Cut-out failure, n(%)	0(0)	-	-	-
Fracture collapse, n(%)	0(0)	-	-	-
Aseptic loosening, n(%)	-	6(12.0)	-	-
DVT, n(%)	2(3.8)	3(6.0)	0.002	0.964
Wound infection, n(%)	1(1.9)	1(1.9)	-	1.000
Cardiovascular complications, n(%)	4(7.8)	2(4.0)	0.138	0.710
Respiratory complications, n(%)	2(3.8)	3(6.0)	0.002	0.964
Urinary complications, n(%)	2(3.8)	1(2.0)	0.001	0.973
Total, n(%)	11(21.2)	16(32)	1.541	0.215

*Abbreviations* CE: cement-enhanced internal fixation, HA: hemiarthroplasty, DVT: deep vein thrombosis

## Discussion

There is a consensus that early surgical treatment of intertrochanteric fractures of the femur allows patients to recover their pre-injury functional status as soon as possible and avoid complications resulting from long-term bed rest [16]. The clinical treatment of elderly osteoporotic patients with intertrochanteric fractures remains challenging. These patients have difficulty returning to their pre-fracture functional level and show poorer outcomes due to reduced osteoporosis, fracture, surgery-related complications, and difficulties with post-operative functional rehabilitation [17]. The best surgical option to treat these patients should be minimally invasive and have fewer postoperative complications [18]. However, there is still no clinical consensus. In our retrospective study, we analyzed the clinical data of the CE and HA groups. We concluded that utilizing the PFNA cement-enhanced technique to treat intertrochanteric fracture in elderly patients could be a better option.

From our study results, we found that the CE group had better operative time, intraoperative blood loss, and intraoperative and postoperative blood transfusion rates than the HA group, which indicated that the PFNA cement augmentation technique retains the advantages of normal PFNA in the treatment of intertrochanteric fractures [19]. This has important implications for improving the prognosis of elderly patients with intertrochanteric femoral fractures because osteoporosis is more common in elderly patients, which results in a more comminuted intertrochanteric fracture pattern. The surgical intervention with HA requires not only performing the femoral head osteotomy but also broaching the medullary repetitively and even repositioning and fixing the great trochanteric fragment, which could be more traumatic for elderly patients as compared with patients with PFNA internal fixation and the reason for the higher intraoperative blood loss and transfusion rates in the HA group than in the CE group. This result is consistent with the results of a previous study demonstrating that PFNA treatment results in a lower blood loss and shorter operation time as compared to HA treatments [20]. Furthermore, although the intraoperative blood loss in the HA group is much more higher than that of the CE group, the HA group still showed an advantage in postoperative weight-bearing time. Based on our research, the two following factors could be involved: (1) patients in the HA group were treated by a distally fixed non-cemented type of prosthesis; these prostheses provide reliable initial stability intraoperatively, which is a unique advantage for early postoperative pain control and early weight-bearing. Contrarily, although the stability of the spiral blade and bone interface was increased by using the bone cement-enhancement technique in the CE group, as a complete comminuted intertrochanteric fracture tends to be more common in elderly patients with osteoporosis. This type of fracture may cause bone fragment micromotion and lead to pain in the early postoperative period after internal fixation. Another factor is that some surgeons still recommend that osteoporotic elderly patients avoid early-weight bearing to prevent the re-displacement of the fracture following PFNA internal fixation, even though the mechanical studies have confirmed the safety of early weight-bearing activities with assisted support after PFNA [21].

Different surgical interventions could lead to different outcomes in the rehabilitation effects. In the present study, even though the HA group was superior to the CE group in the first and third months after surgery, the CE group showed the same outcome in Harris scores, VAS scores, and Barthel Index as the HA group after 6 months post-operatively, which may contribute to preserving the autologous anatomy, such as the posterior capsule, anterior capsule, and short external rotators. These intact soft tissues significantly contribute to the rehabilitation results. In addition to the anterior capsule, the posterior capsule contributes considerably to hip joint stability [22].

Regarding the postoperative complications, in this study, 6 patients in the HA group had postoperative aseptic loosening, but, regarding the other complications, no significant differences were noted between the two groups. This is similar to the results of previous studies that the cement-enhanced PFNA increases the stability of internal fixation and facilitates postoperative functional recovery without increasing the incidence of associated complications [23, 24]. Moreover, we found that the cement-enhancement PFNA construction could provide more stability in the post-op period, which improves the ability of resistance to cutting out and displacement after surgery and improves the biomechanical stability, especially in cases of unstable intertrochanteric fractures; this has been confirmed by a finite element analysis [25]. In the present study, six patients in the HA group were found to have different extents of femoral stem prosthesis loosening and sinking, and the sinking of the femoral prosthesis in these six patients mainly occurred within 3 months after surgery. In the literature, prosthesis aseptic loosening after artificial hip arthroplasty may result from patient osteoporosis, osteolysis, or external factors that cause micromotion of the prosthesis and destabilize the local biomechanical environment [26]. In the present study, all patients in the HA group used a cementless femoral prosthesis. Although the initial mechanical stability was obtained intraoperatively by selecting the appropriate type of femoral stem prosthesis, secondary stability was obtained after the prosthesis gradually sunk during weight-bearing and timely anti-osteoporosis treatment because osteoporosis was not effectively controlled.

In the present study, it is essential to note that treating intertrochanteric fractures with the cement-enhanced PFNA technique requires good repositioning of the intertrochanteric fracture and injecting the proper amount of cement using the correct method to ensure no cement leakage occurs at the fracture site. Therefore, we have tried and modified the cement injection technique to provide a more efficient and optimal distribution of cement in the femoral head during PFNA cement-enhanced internal fixation. Furthermore, in how to effectively control the tip apex distance of the PFNA spiral blade, Linder et al. showed a thorough experimental verification and reported that this operative technique shortens the tip apex distance length by 2 to 3 mm as compared to the standard PFNA technique to ensure homogeneous cement distribution at the tip of the spiral blade of the head and neck [27].

During the study and follow-up period, the utilization of the cement-enhanced PFNA internal fixation technique in the CE group exhibited remarkable biomechanical merits. This technique not only preserved the advantageous features of PFNA, such as reduced trauma, shorter operative duration, decreased intraoperative blood loss, and simplified technical procedure, but also enhanced the cutting resistance of PFNA internal fixation by means of the spiral blade–cement composite. Furthermore, it improved PFNA’s mechanical stability following internal fixation of comminuted intertrochanteric femoral fractures. However, the present study still has some limitations. First, it is a single-center retrospective study with a few cases analyzed; hence, using blind methods is difficult, and the results are inevitably biased. Second, the surgical results may have been biased because different surgeons carried out the operations. Third, the study’s follow-up period was relatively shorter. The long-term effects of the two different procedures are unknown. Thus, prospective analyses and larger samples with long-term follow-up are required to obtain more reliable conclusions.

## Conclusion

In elderly patients with osteoporotic intertrochanteric fractures, the use of PFNA combined with a cement-enhanced internal fixation technique can achieve satisfactory clinical results. This study showed that the CE group has the advantages of shorter operative time, less intraoperative blood loss, and less trauma in elderly patients. Therefore, the cement-enhanced PFNA technique can be recommended for elderly patients with osteoporotic intertrochanteric fractures.

## Data Availability

The data used to support the findings of this study are available from the corresponding author upon request.
